# Neuronavigation assisted percutaneous balloon compression of the gasserian ganglion for trigeminal neuralgia. How I do it

**DOI:** 10.1007/s00701-024-06203-x

**Published:** 2024-07-31

**Authors:** P. Rochat, J. B. Springborg, P. Birkeland, N. Agerlin

**Affiliations:** Department of Neurosurgery, Rigshospitalet, Copenhagen University Hospital, Copenhagen, Denmark

**Keywords:** Trigeminal neuralgia, Balloon compression, Glycerol injection, Percutaneous, Bradycardia, Neuronavigation, Navigation assisted, Surgery, Complications, Limitations

## Abstract

**Background:**

Surgical treatment for trigeminal neuralgia includes percutaneous techniques, including balloon compression, first described in 1983 by Mullan and Lichtor (J Neurosurg 59(6):1007–1012, 6).

**Method:**

Here we present a safe and simple navigation-assisted percutaneous technique for balloon compression, which can also be used for glycerol injection.

**Conclusion:**

The navigation-assisted percutaneous technique for balloon compression for trigeminal neuralgia is a quick and safe treatment for patients not candidates for microvascular decompression.

**Supplementary Information:**

The online version contains supplementary material available at 10.1007/s00701-024-06203-x.

## Article

### Relevant surgical anatomy

Surgical treatment for trigeminal neuralgia includes percutaneous techniques, including balloon compression, first described in 1983 by Mullan and Lichtor [6]. Recent articles have explained different ways to perform navigation-assisted balloon compression for trigeminal neuralgia [3, 4, 7]. The traditional X-ray-guided procedure with a C-arm can be time-consuming and challenging to complete, and there is a learning curve.

Here we present a safe and simple navigation-assisted percutaneous technique for balloon compression, which also can be used for glycerol injection.

### Description of the technique

The procedure is performed using Brainlab® Neuronavigation (Brainlab AG, Munich, Germany). Before the procedure, a thin sliced CT scan of the skull is performed for navigation purposes, and the operative trajectory is planned in Brainlab Elements®.

**Fig. 1:**
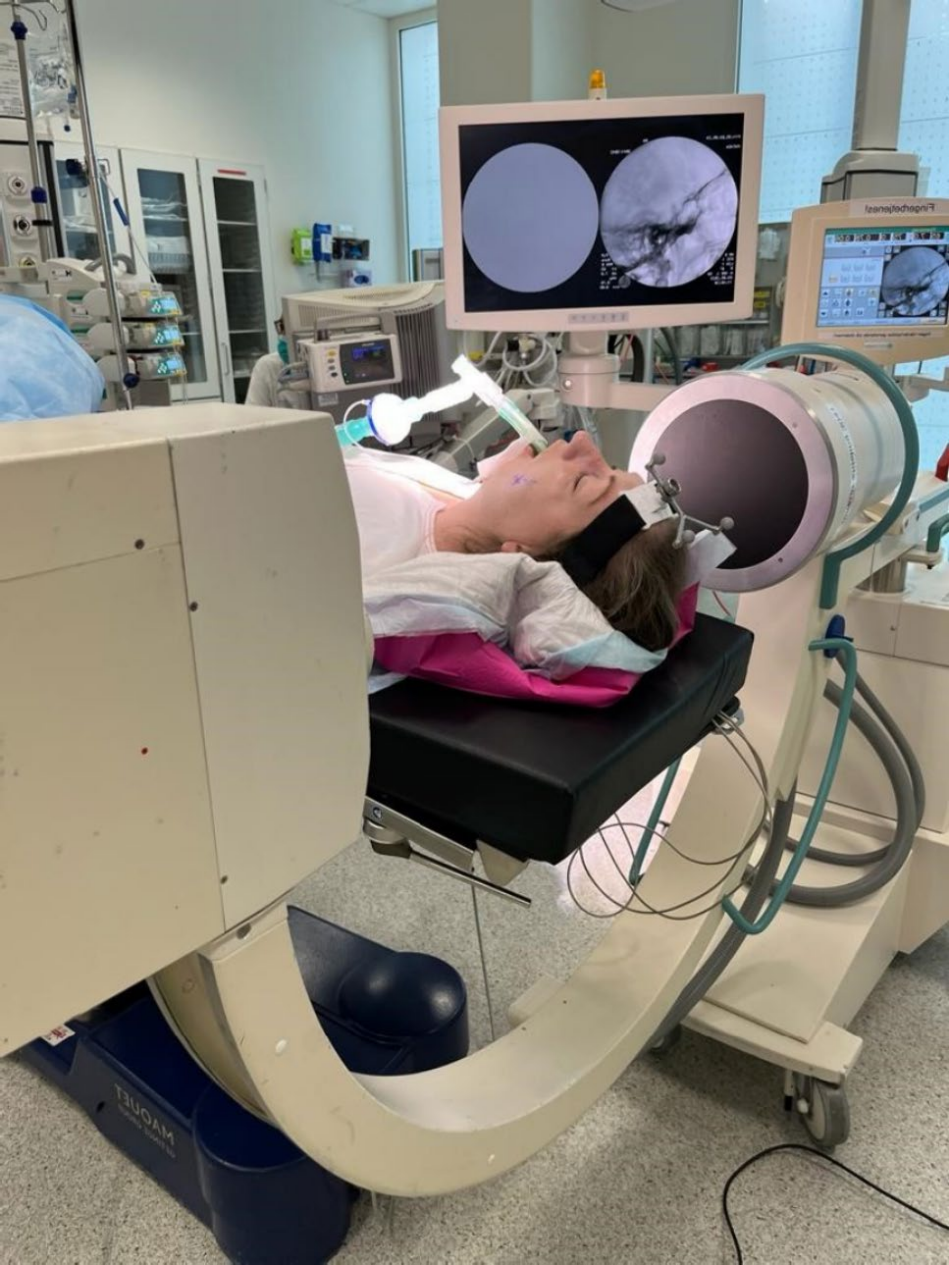
The patient is supine with the head extended approximately 15- 20 degrees. A headband mounted with the BrainLab® reference star is strapped around the forehead

**Fig. 2:**
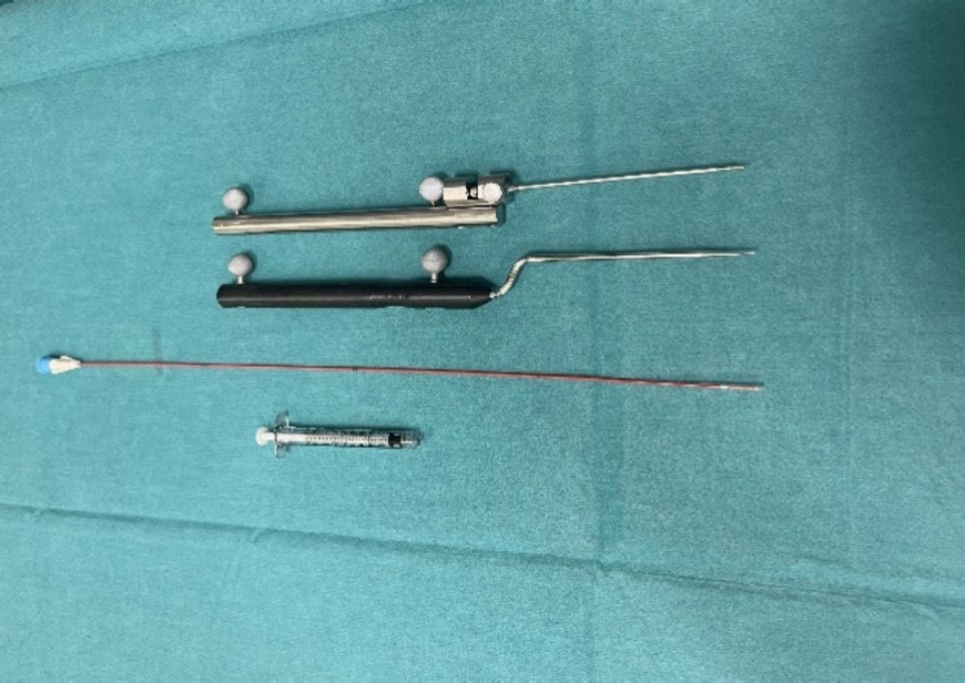
The custom-made handle mounted with the 14 gauche needle is the same length as the BrainLab® pointer. Also shown is the Fogarty® Edwards Lifesciences® 4F catheter with the injection syringe for Omnipaque®

**Fig. 3:**
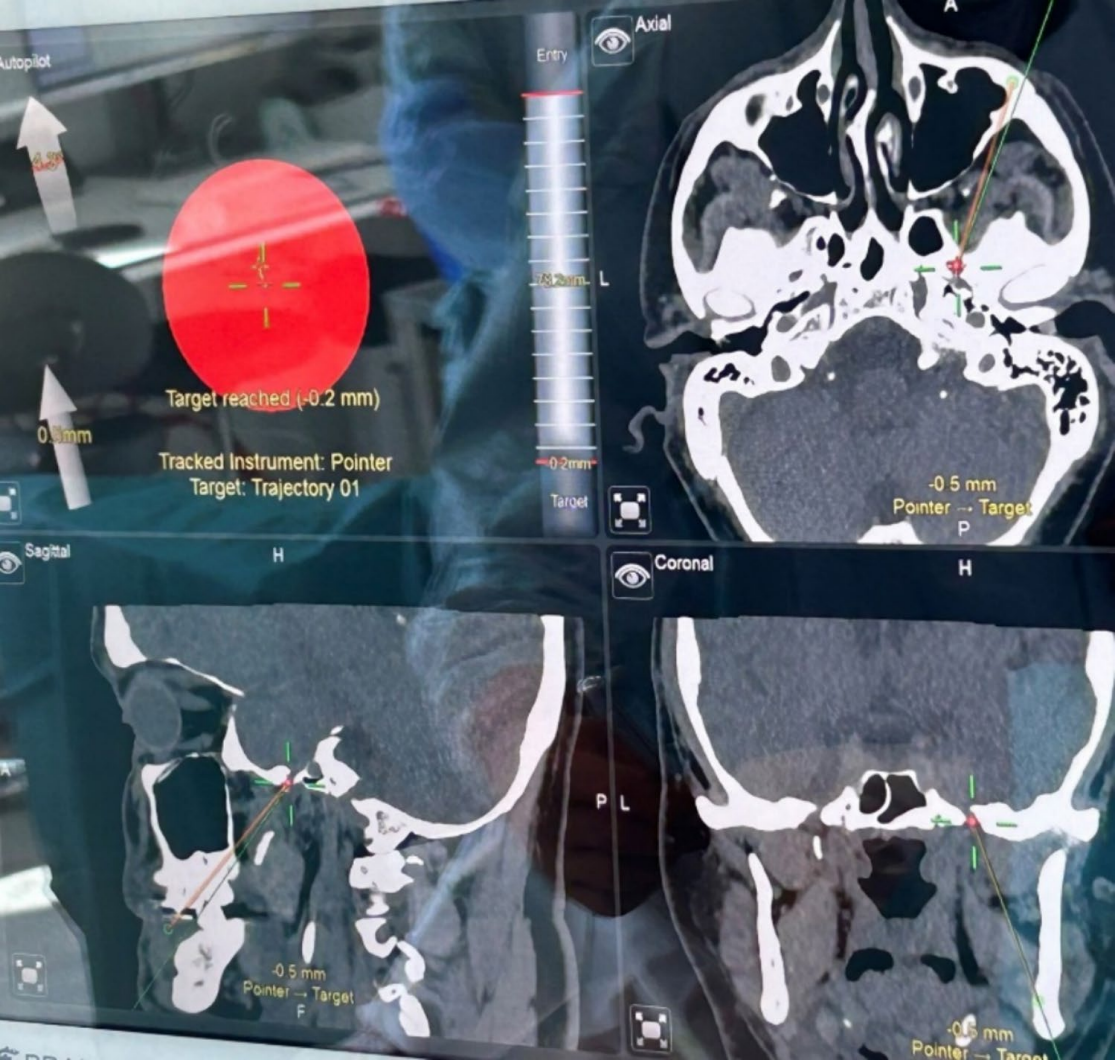
The BrainLab ® screen during the procedure with the “bull's eye” function is shown in the upper left corner

**Fig. 4:**
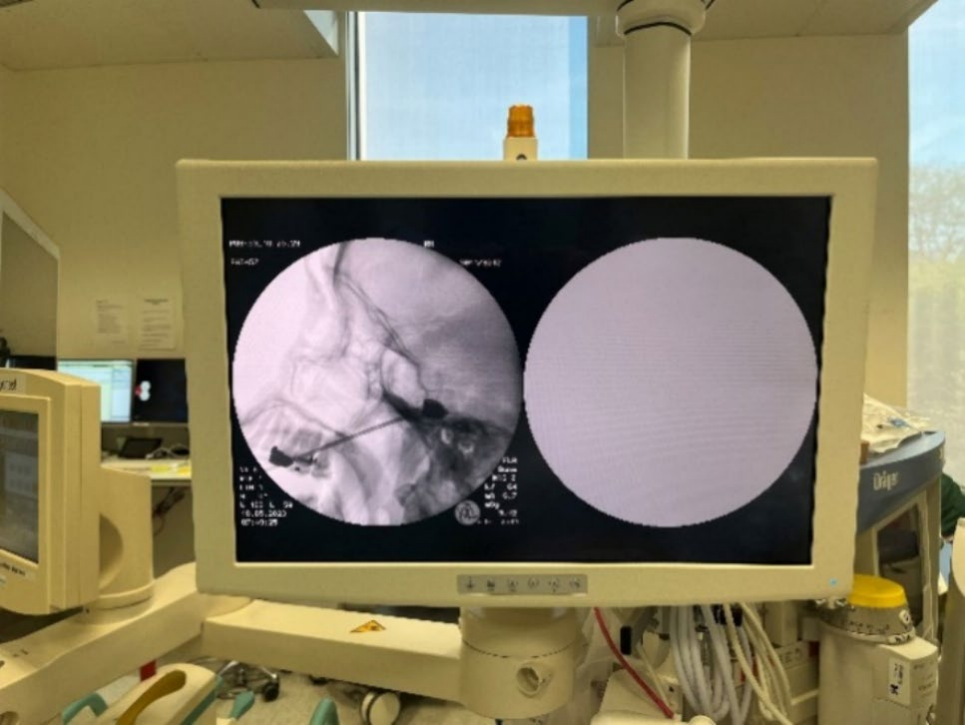
The inserted needle and the balloon filled with contrast medium are seen with fluoroscopy

During surgery, the patient is supine with the head extended approximately 15- 20 degrees (Fig. 1). A headband mounted with the Brainlab® reference star is strapped around the forehead. We use intravenous antibiotic prophylaxis before the operation. To perform the procedure, we use a custom-made CE-approved handle (Pelomi Medical, Albertslund, Denmark), which, mounted with a 14-gauge needle, has the same length as the Brainlab® pointer for navigation (Fig. 2). The entry point is often approximately 1–2 cm lateral to the mouth. The needle is guided through the foramen ovale with neuronavigation using the “bulls’ eye” function (Fig. 3). From a practical perspective, it is always possible to get the needle through the foramen ovale, and fluoroscopy with a C-arm ensures the correct position of the needle. The inner stylet is removed, and a Fogarty® 4F catheter (Edwards Lifesciences, Irvine, CA, USA), produced for intraarterial use, is inserted, and the correct position is confirmed with fluoroscopy (Figs. 2 and 4). The balloon is then filled with approximately 0.8 mL Omnipaque® contrast medium (GE Healthcare, Chicago, Il, USA), and the ganglion is compressed for 2 min (Fig. 4). the balloon must be placed inside the skull to keep it in Meckel´s cave. A “pear shape” is the optimal shape of the balloon, with the tip of the pear pointing towards the porus trigeminus. After two minutes, the balloon and needle are removed.

The patients are observed for 2 h in the recovery room and can go home the same day. If there is instant relieve of pain, we will instruct the patient to reduce the medication against trigeminal neuralgia gradually with one tablet every 5th day, starting with the last introduced drug, if they receive more than one drug. If they can´t quit medication completely, they continue the treatment often with a lover dose with fewer side effects.

### Indications

We use balloon compression as the 1st choice of surgical treatment in patients with trigeminal neuralgia who are not candidates for microvascular decompression (MVD). This includes patients with multiple sclerosis unless they have an apparent neurovascular conflict and absence of brainstem plaques in the relevant area, patients with no MR-verified neurovascular contact, and patients with previously failed MVD where re-operation is not indicated. It can also be offered to some patients with secondary trigeminal neuralgia, where decompression of the trigeminal nerve is impossible.

### Limitations

The procedure is not recommended for patients with trigeminal neuropathy.

### Complications and avoidance of those

The complication rate is low. You may see bradycardia and very seldom short-term cardiac arrest intraoperatively, usually well-treated with atropine. The risk of bradycardia is not seldom, and we give prophylactic treatment with glycopyrroniumbromid 1 mg i.v.

Other potential complications are bleeding, meningitis, double vision [1], mastication weakness, and reactivation of Herpes infection. Numbness in the ipsilateral face indicates good compression and is not considered a complication. It is important to stay extra orally with the needle during the procedure to avoid contamination of the needle from oral mucosa.

### Specific information to the patients

It is essential to inform the patients of potentially permanent numbness in the ipsilateral side of the face and mouth. In the literature the success rate is between 70 to more than 90% [2, 5]. The duration of pain relief is individual but can last for years.

## Supplementary Information

Below is the link to the electronic supplementary material.

Supplementary file1 (MP4 343622 KB)

## Data Availability

Not applicable.
